# Thermal imaging responses of lower-limb muscles following anaerobic testing in male soccer players: A time-series approach

**DOI:** 10.1371/journal.pone.0331102

**Published:** 2025-10-13

**Authors:** Sezgin Korkmaz, Rohit K. Thapa, Nicola Relph, İsmail Çalık, Hüseyin Şahin Uysal

**Affiliations:** 1 Burdur Mehmet Akif Ersoy University, Faculty of Sport Sciences, Burdur, Turkey; 2 Symbiosis International (Deemed University), Symbiosis School of Sports Sciences, Pune, India; 3 Edge Hill University, Ormskirk, Lancashire, United Kingdom; Universidad Complutense de Madrid Escuela Universitaria de Enfermeria Fisioterapia y Podologia, SPAIN

## Abstract

Exercise-induced thermoregulatory responses may vary by sport, yet limited evidence exists on how soccer players respond to high-intensity anaerobic testing. This study aimed to evaluate the acute changes in lower extremity muscle thermal skin temperature(Tsk) in amateur male soccer players following the Wingate anaerobic test and to determine the potential effects of moderators (dominant leg, smoking status, body height, and body fat percentage) on these responses. A total of 26 amateur male soccer players participated in this study, which employed a repeated-measures cross-sectional design. Infrared thermography(IRT) was used to record Tsk data from six anatomical locations(bilateral quadriceps, hamstrings, and gastrocnemii) at five different time points(baseline, 15 seconds, 4, 8, and 12 minutes) following a Wingate anaerobic test. Data were analyzed using the Frequentist and Bayesian repeated-measures ANOVA. The results showed a statistically significant effect of time in the right quadriceps region(*p* = 0.01,ηp² = 0.15,BFincl = 19.51). Post-hoc analysis indicated a significant increase in the Tsk of the right quadriceps at 12 minutes following the test, compared to baseline measurements(*p* = 0.02; BF_10_ = 17.931). Moderator analyses revealed that body fat percentage influenced Tsk responses, particularly in the quadriceps and hamstring regions(*p* = 0.01–0.03,ηp² = 0.25,BFincl = 3.100–3.958). Players with lower body fat showed significantly greater increases in quadriceps and hamstring muscle TSK than players with higher body fat (*p* < 0.05). In conclusion, this study highlights a notable rise in dominant quadriceps skin temperature following high-intensity anaerobic exercise in amateur male soccer players. Body fat percentage appears to modulate these thermal responses, underlining its importance when interpreting IRT results in sports settings. These findings may have practical implications for performance monitoring and thermal recovery strategies in soccer players.

## Introduction

The increasing intensity of competition and individual performance demands in soccer have increased players’ physical and physiological demands [1]. This has necessitated continuous performance monitoring and led coaches to utilize monitoring methods based on physiological responses during matches and training [2,3]. One of the monitoring methods coaches use is infrared thermography (IRT).

IRT has recently become a frequently used technique for assessing skin tissue perfusion [4]. IRT is a diagnostic method based on the ability to record infrared radiation emitted by the skin and can noninvasively indicate physiological or pathological skin temperature changes [5]. Therefore, IRT is the preferred method for monitoring training load and detecting injuries based on skin temperature (T_sk_) responses in various sports, including soccer [6]. This is because abnormal changes in T_sk_ can be an early indicator of conditions such as overload or inflammation [7].

Soccer players exhibit various physiological responses to sport-specific stimuli during competition or training, and one of these responses is thermoregulation [8]. During exercise, the body increases metabolic energy production to support muscle contractions, producing muscle heat [3,9]. This heat is transported to the skin surface via the bloodstream, causing an increase in the T_sk_ [10]. Furthermore, vasodilation occurring during exercise supports this increase [11]. Research has evaluated the increase in Tsk as an inflammatory response, indicating that this increase may persist after intense physical activity [10,12]. Therefore, pre- and post-training Tsk measurements can provide indirect information about muscle damage, recovery, and injury risk [7,13]. Because it is often difficult to directly determine the fatigue levels of soccer players during training or matches, IRT stands out as an essential and practical monitoring tool for coaches and field professionals to visualize these physiological changes.

IRT is a widely used method for assessing skin temperature responses in athletes. But, the existence of various moderators that may influence these assessments must be considered. These moderators can cause Tsk responses to vary according to individual differences. These potentially confounding variables must be included in the analysis process to interpret the data obtained with IRT correctly. For example, a previous study reported a significant negative correlation (*r* = −0.51 to −0.63, *p* = 0.01) between body composition and the T_sk_ of athletes [14]. Another study evaluated the thermal asymmetries of athletes before and after training based on the dominant region and reported that the dominant region had higher T_sk_ compared to the non-dominant region (Cohen’d = 0.20 to 0.50, *p* < 0.05) [15]. In addition, researchers reported that exercise intensity (i.e., mean heart rate 150 and above) [16] and performance test conditions (e.g., Wingate test and treadmill tests) [10] also significantly affected T_sk_. Although many factors influence the Tsk of athletes, each of these factors has been investigated in different sports (e.g., sailing, futsal, track and field) [10,14,15].

In the relationship between IRT and soccer, researchers have focused on monitoring fatigue and training load [12]. However, T_sk_ responses of soccer players based on performance test conditions have been evaluated in limited studies [1,12]. One study examined the effects of a soccer simulation protocol applied for 45 and 90 minutes on the T_sk_ of soccer players [12]. The other study focused on the T_sk_ responses of the lower extremities of soccer players before and after a speed test [1]. Although the T_sk_ responses of athletes are influenced by various factors [10,14–16], previous studies did not address the regulatory factors affecting the T_sk_ responses of soccer players such as smoking status, height, and fat percentage [1,12]. Furthermore, the T_sk_ responses of soccer players have not yet been evaluated using an anaerobic-based test protocol, such as the Wingate test. Similarly, few studies have examined T_sk_ responses in athletes before and after the Wingate test [10,17]. Therefore, original studies examining T_sk_ responses and the effects of regulatory factors on soccer players after anaerobic testing are needed.

Understanding the Tsk responses during intense efforts specific to soccer and identifying the regulatory factors that influence these responses are crucial for revealing soccer players’ thermoregulatory responses, supporting their on-field performance, and addressing a significant gap in the literature with an original approach. This study aimed to evaluate amateur male soccer players’ acute lower-limb muscle T_sk_ responses to anaerobic test conditions and identify potential moderators’ effects on these acute responses. It was hypothesized that the anaerobic-based Wingate test protocol could increase T_sk_ of the lower extremity muscles in male amateur soccer players and that moderator variables could be effective in this increase.

## Methods

### Study design

This study was a double-blind, repeated measures cross-sectional study. Blinding was performed in the following three stages: (i) Participants were informed about the protocol but not the purpose of the study, (ii) The researcher who recorded the data was blinded for statistical analysis, and (iii) The researcher who performed the data analysis was blinded to the data collection process. The Strengthening the Reporting of Observational Studies in Epidemiology (STROBE) criteria were followed [18]. While collecting T_sk_ data using IRT, the Thermographic Imaging in Sports and Exercise Medicine (TISEM) checklist was used to reduce the risk of bias and standardize the analyses [4]. This study was registered in the Open Science Framework (OSF) (DOI: https://doi.org/10.17605/OSF.IO/JW89Q). The details of the checklists used for this study are presented in S1 Appendix. In addition, details of the study files are provided with open access through the OSF (https://osf.io/qfhvd/).

### Participants

Thirty-one active male soccer players from the amateur league in Burdur Province, Turkey, participated in the study. These players were soccer players who competed in official matches once a week and trained four days a week (age: 20.07 ± 1.14 years; height: 177.80 ± 6.07 cm, weight: 74.52 ± 9.31 kg, body fat percentage: 16.89 ± 5.37; body mass index: 20.07 ± 1.14 kg/m^2^). The following inclusion criteria were used to select participants: (i) no injury in the last six months, (ii) no chronic health problems, (iii) no infectious health problems (flu, cold, etc.) that could affect skin temperature at least two weeks before the study protocol, (iv) actively playing soccer in amateur soccer leagues, (v) between the ages of 18–25, and (vi) healthy young male soccer players.

The results from previous studies were used to determine the sample size [10,17]. A *priori* power analysis was performed using G*Power software (version 3.1, University of Düsseldorf, Germany) (ANOVA: Repeated measures, between factors, α = 0.05, β = 0.80, Cohen’s d = 0.70, number of groups = 5, number of measurements = 5, and *r*-value among repeated measures = 0.5). The minimum sample size was 20 participants, and 31 male amateur soccer players were included in this study. Recruitment began and ended on 24.04.2024. The participants were informed of the study’s positive and negative aspects, and the study’s aims were kept confidential to prevent participation bias. Participation was voluntary, participants provided verbal consent and were allowed to withdraw from the study at any time. This study was conducted with the approval of the Burdur Mehmet Akif Ersoy University Non-Interventional Clinical Research Ethics Committee (Code: 2024/10), and ethical principles were taken into account following the Declaration of Helsinki.

### Experimental design

**Fig 1:**
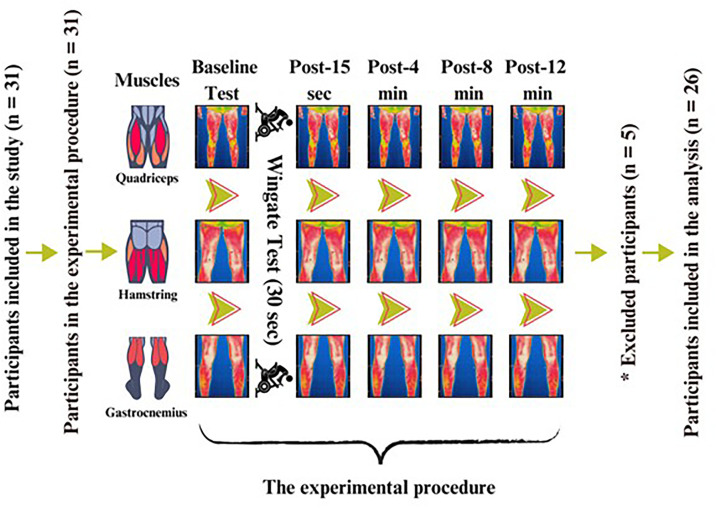
The study design based on repeated measures. ***Legend.*** sec: Seconds; min: Minutes. *: Participants’ data were excluded from the analysis because thermal images were obtained incorrectly based on athletes’ sweating.

This study was conducted in the Faculty of Sports Sciences performance laboratory at Burdur Mehmet Akif Ersoy University, Turkey. The temperature and humidity of the laboratory environment were standardized at threshold values (22 ± 2 °C; φ = 40–60%) recommended by previous research [4] and isolated from devices that create air currents (e.g., ventilation and air conditioning). The participants waited to acclimate to the test environment for 20 minutes [4] by sitting or standing depending on their preference. The participants were instructed not to eat large meals, consume caffeine, or drink alcohol and to avoid Ultraviyole (UV) rays for 4 hours before the measurement. The study was conducted 48 hours after a training session. The participants did not participate in any physiotherapy sessions prior to the testing. The tests were conducted in the afternoon (between 1.00 pm and 3.00 pm). The details of the experimental design are shown in Fig 1.

### Measurements

#### Anthropometric measurements.

Bioelectrical impedance analysis technology (InBody 270, Biospace, California, USA) was used to assess participants’ body composition. This measurement has excellent reliability (ICC = 0.98 to 1.00) and validity (*r* = 0.97 to 0.99) [19]. Participants were instructed to refrain from eating, drinking, or exercising for at least 2–3 hours before measurement to minimize changes in hydration status and body water distribution. Measurements were performed barefoot and in light clothing.

#### Dominant Leg Test.

The ball-kicking test was performed to determine the dominant leg of the participants. The participant was instructed to kick a stationary ball placed on the ground. The leg they naturally preferred to kick was considered the dominant leg of the participants [20].

#### Wingate cycle ergometer test.

The Wingate cycle ergometer test was performed on a stationary bicycle of a Peak Bike Ergometric 894E (Monark Exercise AB, Vansvro, Sweden). A 5-minute warm-up was performed on a bicycle ergometer at low resistance (approximately 50 watts) to prepare participants for maximum effort. Participants completed the test at maximum effort for 30 seconds against a resistance set at 7.5% of their body mass [21].

#### Skin temperature measurements.

An FLIR thermographic camera (FLIR T530, FLIR Systems Inc., Wilsonville, OR, USA) was used to assess T_sk_ with IRT. This thermal camera had a sensor array size of 320 × 240 pixels and a thermal sensitivity of <40 mK. The device can obtain thermal images with an accuracy of ±2% in the spectral range of 7.5 to 14.0 µm [22].

**Fig 2:**
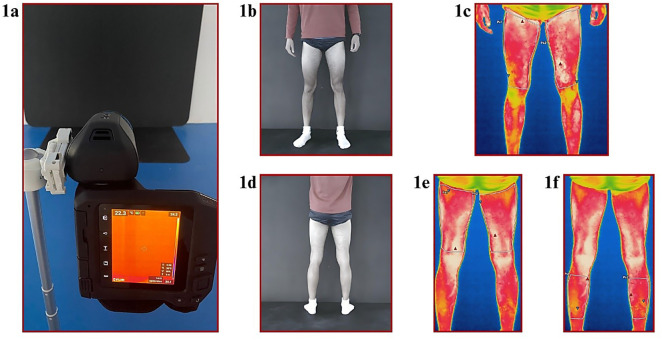
Thermal skin temperature measurement and data extraction process. ***Legend.* 1a**: Measurement environment; **1b**: Anterior image obtained in the measurement environment; **1c**: Thermographic anterior image obtained in the measurement environment and the quadriceps region of interest; **1d**: Posterior image obtained in the measurement environment; **1e**: Thermographic posterior image obtained in the measurement environment and the hamstrings region of interest; **1f**: Thermographic anterior image obtained in the measurement environment and the gastrocnemius region of interest.

The emissivity of the camera was set to 0.98 to obtain thermal images. Measurements were obtained from a distance of two meters using a tripod at a height of one meter. Before the measurements of each participant, the automatic laser alignment feature of the FLIR thermographic camera was preferred to calibrate camera and minimize the error margin [4]. The environment in which the thermal image was obtained was free from artificial or natural UV sources, and the images were obtained on a dark background. The details of the skin temperature measurements are shown in Fig 2.

#### Determining regions of interest and data extraction.

The images recorded with the thermal camera were stored in the FLIR Ignite™ cloud account. Data were processed with FLIR Thermal Studio Pro® (Teledyne FLIR LLC, United States) software. The following six regions of interest (ROIs) were evaluated in the study [10]: (i) right quadriceps, (ii) left quadriceps, (iii) right hamstring, (iv) left hamstring, (v) right gastrocnemius, (vi) left gastrocnemius. The procedures suggested by the researchers were considered for ROI selection and T_sk_ measurements [4]. Each ROI was manually delineated based on standardized anatomical landmarks: quadriceps (from the anterior superior iliac spine to the superior patella), hamstrings (from the ischial tuberosity to the popliteal crease), and gastrocnemius (point between the popliteal crease and Achilles tendon) [23]. Although the polygonal area varied according to individual limb dimensions, the same trained researcher performed all delineations using consistent anatomical reference points to ensure standardization and reproducibility across participants. The mean T_sk_ within this polygon was used for statistical analysis. A soft, clean, dry towel was used to remove excess moisture from the athletes’ skin. However, the thermal images of some participants were affected because the participants continued to sweat during the measurements. Therefore, data of 5 participants were not included in the analysis. Data extraction procedures were performed by a researcher who was not involved in the statistical analysis. Thermal images were excluded from analysis if excessive sweating led to visible blurring, reflection artifacts, or temperature distortion in the ROIs. This exclusion was guided by stringent, predefined criteria from established thermographic standards [4]. An experienced thermography analyst, blinded to the study hypotheses, meticulously conducted the assessment to ensure objective evaluation. Consequently, this rigorous quality control procedure excluded five participants, reinforcing the integrity and reliability of our study findings.

### Statistical analysis

T_sk_ values were obtained from six ROIs at five different times. Four potential moderator variables affecting the T_sk_ responses of athletes (smoking status, dominant leg, body height status, and body weight status) were determined based on the results of a previous study [4]. When creating subgroups for variables related to body composition, references suggested by researchers were considered [24,25]. For body height, participants were categorized into three groups [25]: (i) 174 cm and under, (ii) 175–179 cm, and (iii) 180 cm and above. For body fat percentage, classifications were made according to categories suggested researchers [24]: 5–10% (Athletic), 11–14% (Good), 15–20% (Acceptable), 21–24% (Overweight), and above 24% (Obese).

One-way repeated measures analysis of variance (ANOVA) was performed to analyze the T_sk_ responses of the muscle groups based on time. Within-subject effects between time and moderator variables and between-subject effects of differences between moderator groups were determined using two-way repeated measures ANOVA. The normality distribution of the data was evaluated using the Shapiro-Wilk test to ensure assumptions. Data were assumed to be normally distributed and reported using descriptive statistics (i.e., mean and standard deviation). The homogeneity of variances was analyzed using the Levene test, whereas potential outliers were analyzed using a box plot. The assumption of sphericity was tested using the Mauchly test, and the Greenhouse-Geisser correction was applied to cases of violation of sphericity. Post-hoc pairwise comparisons were performed with Bonferroni correction if statistically significant differences were observed. Partial eta squared (ηp2) was used to calculate the effect size, which was interpreted based on the following references [26]: 0.01 = small effect, 0.06 = medium effect, and 0.14 = large effect. The significance level was set at *p* < 0.05 in the statistical analyses.

Researchers have reported that Bayesian statistics may offer more flexible and adaptable analyses in small sample sizes or complex models, and provide the opportunity to comment directly on the probability distributions of the parameters [27]. Therefore, in addition to the frequentist (i.e., probabilistic significance tests) approach, the data were analyzed using the Bayesian approach. Bayesian repeated measures ANOVA (Bayesian RM-ANOVA) was used to analyze the data. The results were reported using the Bayes factor for hypothesis testing (BF_10_) and the Bayes factor for inclusion in the model comparison (BF_incl_). BF_10_ quantifies the relative evidence for one hypothesis over another (e.g., H0 compared to H1), while BF_incl_ expresses the contribution of a variable to the model. The strength of evidence for hypothesis H1 of BF_10_ and BF_incl_ was interpreted based on the following references [28]: extreme (BF_10_ and BF_incl_ = > 100), very strong (BF_10_ and BF_incl_ = 30–100), strong (BF_10_ and BF_incl_ = 10–30), moderate (BF_10_ and BF_incl_ = 3–10), and anecdotal (BF_10_ and BF_incl_ = 1–3). For the strength of evidence for hypothesis H0, the following references were considered [28]: extreme (BF_10_ and BF_incl_ = > 0.001), very strong (BF_10_ and BF_incl_ = 0.001–0.03), strong (BF_10 _= 0.03–0.01), moderate (BF_10_ and BF_incl_ = 0.01–0.3), and anecdotal (BF_10_ and BF_incl_ = 0.3–1). The use of Bayesian inference complemented traditional analysis by reducing sensitivity to small sample sizes, outliers, and violations of normality assumptions. This dual approach allowed us to confirm key findings through converging evidence across both statistical paradigms. Statistical analyses were performed using JASP (version 0.19.1.0, Amsterdam, Netherlands), and Origin Pro (version 2025, Origin Lab Co., USA) was used for data visualization.

## Results

### Adherence to intervention

Thirty-one amateur male soccer players completed the study protocol. No injuries occurred during the study. Five participants continued to sweat during the tests, resulting in inaccurate thermal images. Therefore, data from five participants was not included in the analysis.

### Quadriceps muscles

The main effect of time on the right quadriceps was statistically significant, providing strong evidence supporting hypothesis H1 (*p* = 0.01, ηp² = 0.15, BF_incl_ = 19.51). Post-hoc pairwise comparisons revealed statistically significant differences between the measurements taken 12 minutes after the Wingate and baseline tests (*p* = 0.02; BF_10 _= 17.931).

**Fig 3:**
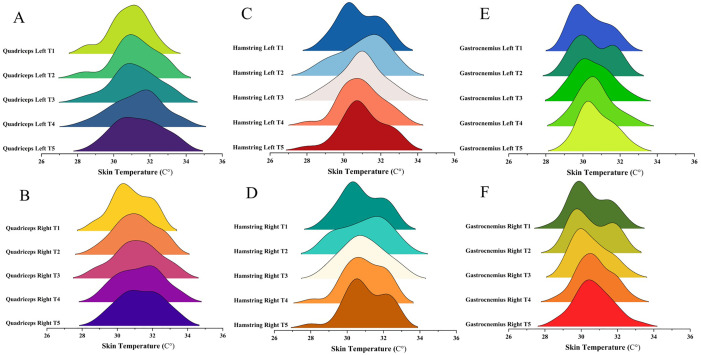
Skin temperature responses of amateur male soccer players to different conditions. ***Legend.*** T1: Baseline test before wingate cycle ergometer test; T2: 15 seconds after wingate cycle ergometer test; T3: 4 minutes after wingate cycle ergometer test; T4: 8 minutes after wingate cycle ergometer test; T5: 12 minutes after wingate cycle ergometer test; A: Left quadriceps muscle; B: Right quadriceps muscle; C: Left hamstring muscle; D: Right hamstring muscle. Each curve represents a specific time point. The curve’s height indicates the number of measurements made at specific temperature intervals. Higher curves indicate more data points at that temperature. These graphs visualize the skin temperature distribution and mean values for the relevant muscle group at each time point.

In contrast, no significant time effect was observed for the left quadriceps (*p* = 0.05, ηp² = 0.10; BF10 = 1.00, BFincl = 1.63). Similarly, interaction effects with moderator variables were not significant for either muscle (*p* = 0.14 to 0.83; BF_10_ = 0.065 to 0.407; BF_incl_ = 0.143 to 1.257). While descriptive data are presented in S2 Appendix, details of the results are presented in Table 1. Additionally, details regarding muscle Tsk distributions over time for each muscle group are presented in Fig 3.

**Table 1:** The results for main and interaction effects on T_sk_ responses of amateur male soccer players.

Variables	Probabilistic RM-ANOVA								Bayesian RM-ANOVA					
	Time main effect				Time x Smoke interaction (*p*-value)	Time x DL interaction (*p*-value)	Time x BH interaction (*p*-value)	Time x BF interaction (*p*-value)	Time main effect		Time x Smoke interaction BF_10_ (BF_incl_)	Time x DL interaction BF_10_ (BF_incl_)	Time x BH interaction BF_10_ (BF_incl_)	Time x BF interaction BF_10_ (BF_incl_)
	*df*	*F-value*	*p-*value	η_p_^2^					BF_10_	BF_incl_				
Left Quadriceps	2.46	2.94	0.05 ^a^	0.10	0.43	0.22	0.31	0.48	1.00	1.642	0.094 (0.143)	0.425 (0.536)	0.168 (0.254)	0.111 (0.337)
Right Quadriceps	3.10	4.73	0.01*^ab^	0.15	0.83	0.29	0.94	0.36	1.00	19.51	0.065 (0.156)	0.275 (0.567)	0.070 (0.173)	0.407 (1.257)
Left Hamstring	2.20	0.73	0.49 ^a^	0.02	0.23	0.14	0.39	0.58	0.077	0.077	0.020 (0.046)	0.044 (0.093)	0.008 (0.018)	0.009 (0.023)
Right Hamstring	2.43	0.71	0.51 ^a^	0.02	0.27	0.08	0.14	0.63	0.074	0.074	0.013 (0.030)	0.058 (0.120)	0.028 (0.066)	0.004 (0.011)
Left Gastrocnemius	1.99	1.38	0.25 ^a^	0.05	0.64	0.53	0.63	0.51	0.190	0.190	0.012 (0.027)	0.015 (0.034)	0.012 (0.023)	0.012 (0.026)
Right Gastrocnemius	2.76	0.56	0.62 ^a^	0.02	0.53	0.51	0.40	0.66	0.06	0.06	0.005 (0.012)	0.004 (0.010)	0.004 (0.010)	0.002 (0.006)

Legend RM-ANOVA: Repeated measures ANOVA*;* DL: Dominant leg; BH: Body height; BF: Body fat; BF_10_: Bayes factor 10; BF_incl_: Bayesian inclusion factor. a: Green-house Geisser corrections were performed for sphericity assumptions were violated; b: The statistically significant difference in favor of Post-12 min compared to baseline; *: significantly different between time.

### Hamstring muscles

No significant time effects were found for the left or right hamstring (*p* = 0.49 to 0.51, ηp² = 0.02; BF_10_ = 0.074 to 0.077, BF_incl_ = 0.074 to 0.077). In addition, interaction effects with moderator variables were also non-significant (*p* = 0.08 to 0.63; BF_10_ = 0.004 to 0.058, BF_incl _= 0.011 to 0.120). While descriptive data are presented in S2 Appendix, details of the results are presented in Table 1. Additionally, details regarding muscle Tsk distributions over time for each muscle group are presented in Fig 3.

### Gastrocnemius muscles

No significant time effects were found for either gastrocnemius muscle T_sk_ (*p* = 0.25 to 0.62, ηp² = 0.02 to 0.05; BF_10_ = 0.190 to 0.060, BF_incl_ = 0.060 to 0.190). Similarly, interaction effects between time and moderator variables were non-significant (*p* = 0.40 to 0.66; BF_10_ = 0.002 to 0.015, BF_incl_ = 0.006 to 0.034). While descriptive data are presented in S2 Appendix, details of the results are presented in Table 1. Additionally, details regarding muscle Tsk distributions over time for each muscle group are presented in Fig 3.

### Moderator analysis

Statistically significant differences were observed when evaluating the T_sk_ responses of the quadriceps and hamstrings about body fat ratio groups (*p* = 0.01 to 0.03, ηp² = 0.25 to 0.40; BF_incl_ = 3.100 to 3.958). Post-hoc analyses revealed that participants with a body fat percentage of 24% or higher exhibited lower T_sk_ responses, with statistically significant differences compared to the other groups (Left quadriceps: [compared to 5–10% body fat: *p* = 0.01; BF_10_ = 2.166 x 10^+6^], [compared to 11–14% body fat: *p* = 0.02; BF_10_ = 46143.774], [compared to 15–20% body fat: *p* = 0.02; BF_10_ = 2.088 x 10^+9^]; Right quadriceps: [compared to 5–10% body fat: *p* = 0.01; BF_10_ = 635142.879]; Left hamstring: [compared to 5–10% body fat: *p* = 0.01; BF_10_ = 5.993 x 10^+8^], [compared to 11–14%: *p* = 0.03; BF_10_ = 12509.541]; Right hamstring: [compared to 5–10% body fat: *p* = 0.02; BF_10_ = 4.189 x 10^+6^]). These results emphasized that participants with lower body fat percentages exhibited higher T_sk_ responses than those with higher percentages.

On the other hand, no significant between-group differences were found for smoking status, dominant leg, or body height across any muscle group (*p* = 0.09 to 0.98, ηp² = 0,00–0,07; BF_10_ = 0.026 to 0.793; BF_incl_ = 0.257 to 0.741). Similarly, body fat ratio did not significantly affect gastrocnemius Tsk responses (*p* = 0.26 to 0.40, ηp² = 0.00 to 0.06; BF_10_ = 0.406 to 0.569; BF_incl _= 0.272 to 0.381). Details of the moderator group comparisons based on between-subject effects are presented in Table 2. In addition, descriptive statistics of the moderator groups are presented in S3 Appendix.

**Table 2:** Results for between-subject effects of moderator variables.

Variables/Moderator factors	Probabilistic RM-ANOVA		Bayesian RM-ANOVA	Probabilistic RM-ANOVA		Bayesian RM-ANOVA	Probabilistic RM-ANOVA		Bayesian RM-ANOVA		Probabilistic RM-ANOVA		Bayesian RM-ANOVA
	Smoke status			Dominant leg status			Body height status				Body fat status		
	*F(df)*	*p-value (*η_p_^2^)	BF_10_ (BF_incl_)	*F(df)*	*p-value (*η_p_^2^)	BF_10_ (BF_incl_)	*F(df)*	*p-value (*η_p_^2^)	BF_10_ (BF_incl_)	*F(df)*	*p-value (*η_p_^2^*)*	BF_10_ (BF_incl_)	
Left Quadriceps	0.32 (1,24)	0.57 (0.00)	0.375 (0.443)	2.98 (1,24)	0.09 (0.07)	0.607 (0.927)	1.17 (2,23)	0.32 (0.01)	0.387 (0.494)	5.36 (4,21)	0.01*^a^ (0.40)	0.676 (3.958)	
Right Quadriceps	0.00 (1,24)	0.98 (0.00)	0.029 (0.423)	2.01 (1,24)	0.16 (0.03)	0.048 (0.741)	0.41 (2,23)	0.66 (0.00)	0.026 (0.385)	4.81 (4,21)	0.01*^b^ (0.37)	0.052 (3.433)	
Left Hamstring	0.00 (1,24)	0.96 (0.00)	0.605 (0.418)	1.35 (1,24)	0.25 (0.01)	0.781 (0.544)	0.79 (2,23)	0.46 (0.00)	0.674 (0.454)	4.76 (4,21)	0.01*^c^ (0.35)	1.000 (3.503)	
Right Hamstring	0.00 (1,24)	0.94 (0.00)	0.606 (0.412)	1.25 (1,24)	0.27 (0.01)	0.793 (0.563)	0.52 (2,23)	0.59 (0.00)	0.569 (0.397)	3.21 (4,21)	0.03*^d^ (0.25)	1.000 (3.100)	
Left Gastrocnemius	0.03 (1,24)	0.84 (0.00)	0.490 (0.336)	0.21 (1,24)	0.64 (0.00)	0.513 (0.349)	1.53 (2,23)	0.23 (0.03)	0.751 (0.508)	1.42 (4,21)	0.26 (0.06)	0.569 (0.381)	
Right Gastrocnemius	0.33 (1,24)	0.57 (0.00)	0.514 (0.346)	0.00 (1,24)	0.95 (0.00)	0.457 (0.307)	0.47 (2,23)	0.63 (0.00)	0.382 (0.257)	1.06 (4,21)	0.40 (0.00)	0.406 (0.272)	

*Legend* *: significantly different between time.; RM-ANOVA: Repeated measures ANOVA*;* DL: Dominant leg; BH: Body height; BF: Body fat; BF_10_: Bayes factor 10; BF_incl_: Bayesian inclusion factor; a: Post-hoc analyses indicated the following significant results; (i) The statistically significant difference in favor of 5–10% compared to body fat ratio of 24% and above, (ii) The statistically significant difference in favor of 11–14% compared to body fat ratio of 24% and above, (iii) The statistically significant difference in favor of 15–20 compared to body fat ratio of 24% and above; b: Post-hoc analyses indicated the statistically significant difference in favor of 5–10% compared to body fat ratio of 24% and above; c: Post-hoc analyses indicated the following significant results; (i) The statistically significant difference in favor of 5–10% compared to body fat ratio of 24% and above, (ii) The statistically significant difference in favor of 11–14% compared to body fat ratio of 24% and above; d: Post-hoc analyses indicated the statistically significant difference in favor of 5–10% compared to body fat ratio of 24% and above.

## Discussion

This study hypothesized that the anaerobic-based Wingate test could acutely increase amateur male soccer players’ lower body muscle temperature and that this T_sk_ increase would differ among moderator factors. The results confirmed the hypotheses and showed a statistically significant difference in the right quadriceps T_sk_ of amateur male soccer players after the Wingate test compared to the baseline test before the Wingate at the 12th minute, providing strong evidence in favor of hypothesis H1. Furthermore, it was revealed that the body fat ratio affected the T_sk_ responses of male amateur soccer players, and soccer players with lower fat ratios had higher T_sk_ responses than those with higher fat ratios. While no statistically significant differences were found for other muscle groups and moderator variables, the frequentist statistical results were consistent with the Bayesian outcomes. In practical terms, this means that when *p*-values indicated non-significant results, the Bayesian analysis similarly provided weak or anecdotal evidence, suggesting limited support for the alternative hypothesis. Therefore, both approaches highlighted that only the right quadriceps and body fat effects were supported by meaningful statistical evidence.

The right quadriceps exhibited a uniquely significant thermal response among all observed regions, highlighting its predominant involvement during anaerobic exertion. Furthermore, body fat percentage emerged as a key moderator, systematically influencing thermal recovery across major muscle groups. Although the Wingate test does not mimic the technical movements of soccer, it reflects the short-duration, high-intensity efforts frequently performed in the game (i.e., sprints, quick accelerations, and repeated anaerobic activities) [29]. Therefore, it offers a controlled and valid model to examine the thermoregulatory and physiological responses of soccer players under intense anaerobic load. Therefore, this result may primarily be attributed to the muscle activations observed during the Wingate test. Previous studies have indicated that positioning the Wingate bicycle seat post more perpendicular to the ground enhances quadriceps activation during the test (*p* = 0.001 to 0.03) [30] while simultaneously reducing hamstring activation (*p* = 0.03, power = 0.70) [31]. This study conducted the Wingate test with the saddle angled approximately 90 degrees to the ground. The increase in quadriceps muscle activation during the Wingate test may elevate the metabolic energy demand in the targeted region [3]. Consequently, the chemical energy released may have been converted into heat [10], increasing T_sk_ in the area through the circulatory system. Also, the findings may be linked to muscle microtraumas and inflammatory responses occurring in the quadriceps muscle during the Wingate test. A recent study investigated the changes in contraction properties of the quadriceps, hamstring, and gastrocnemius muscles during the Wingate test [32]. The researchers found that the Wingate test protocol resulted in increased stiffness (*p* = 0.03, *r* = 0.47) and a delayed response to stimuli (*p* = 0.00 to 0.02, *r* = 0.51 to 0.66) in the quadriceps muscle [32]. The physiological parameters from another study confirmed these findings, indicating that the Wingate test protocol elevated levels of inflammatory cytokines, including interleukin-6, interleukin-10, and tumor necrosis factor-alpha [33]. Consequently, the statistically significant increase in quadriceps T_sk_ may have resulted from an inflammatory response to muscle damage [11]. Furthermore, the elevated T_sk_ in the right quadriceps may be associated with increased lactate levels during the Wingate test. While the Wingate test is known to elevate lactate levels [34], researchers have reported a moderate positive correlation (*r* = 0.43 to 0.48, *p* < 0.05) between lactate levels and T_sk_ [14]. Many previous studies have confirmed the current study’s findings, indicating that the Wingate test protocol increases T_sk_ in the quadriceps [10,35].

Another notable finding in our study was the inconsistent increase in T_sk_ in the quadriceps muscles. While the time-dependent T_sk_ increase in the right quadriceps was statistically significant, the time-dependent T_sk_ increase in the left quadriceps was non-significant. Considering that 19 out of 26 participants included in the analysis were right-leg dominant, these results can be explained by the dominant and non-dominant leg status. The dominant leg requires more oxygen and nutrients during exercise [36]. This situation can trigger vasodilation in the relevant region and provide blood circulation to be transferred to the skin surface [11]. Therefore, a statistically significant difference may have emerged for the right quadriceps compared to the left quadriceps. In addition, mechanisms specific to inflammatory responses may have triggered the right quadriceps T_sk_ increase [32,33]. The opposite results in quadriceps T_sk_ may also be related to statistical power. Only seven soccer players included in the analysis were left-leg dominant. In studies that are not performed with sufficient sample size, an existing difference may not be reflected in the analysis results [37]. The results of the time main effect for the left quadriceps T_sk_ supported this assumption (*p* = 0.05; BF_incl_ = 1.642).

On the other hand, no significant Tsk changes were observed in the hamstring and gastrocnemius muscles, except for the right quadriceps, during the anaerobic Wingate test. One possible explanation could be that these muscle groups are less active than the quadriceps during seated anaerobic cycling exercises such as the Wingate test. Previous EMG-based studies have shown lower activation levels in the hamstring and gastrocnemius muscles during the Wingate test, particularly when the saddle height is approximately 90° [31]. Furthermore, these muscles may exhibit lower regional metabolic activity and blood flow redistribution under such exercise conditions, which could lead to reduced peripheral heat transfer and, consequently, lower Tsk responses [32]. Alternatively, it is plausible that the gastrocnemius muscle’s thermal inertia and fatty tissue insulation may delay or suppress sudden Tsk increases. Therefore, these regions’ lack of significant temperature changes may reflect exercise-specific biomechanical function and regional thermal physiology.

This study evaluated the acute effects of time and moderator variables on T_sk_ for the first time in amateur male soccer players. This study’s most significant moderator analysis finding was that body fat percentage had a significant moderating effect on Tsk responses across muscle groups. The statistically significant difference between the body fat ratio groups can be attributed to the skin thickness of the participants because the results determined that the T_sk_ of the groups with a higher fat ratio were lower than those with a lower fat ratio. Many previous studies reported that skin thickness affected T_sk_ and that there was an inverse correlation (*r* = −0.43 to −0.68) between the human body mass index and T_sk_ responses [14,38,39]. On the other hand, the results indicated no interaction effect of moderator variables on time concerning acute lower body T_sk_ measurements before and after the Wingate test. The T_sk_ responses of the moderator groups exhibited a parallel trend of increase or decrease throughout the measurements. Similarly, in comparisons of moderator groups based on between-subject effects, no statistically significant results were found for dominant leg status (left leg vs. right leg), smoking status (smoker vs. non-smoker), and body height status (174 cm and under vs. 175 cm to 179 cm vs. 180 cm and above). While taller individuals with a larger surface area may dissipate heat more rapidly than shorter individuals [40], the frequency of leg use during activity may influence skin temperature [41]. Moreover, the vasoactive effects of smoking may impact blood circulation [42], potentially leading to changes in skin temperature. The complex nature of the human thermoregulation system and the heterogeneous structure of the moderator groups may explain the nonsignificant results regarding smoking status, dominant leg status, and body height status. For instance, while smoking status may affect T_sk_ in a shorter participant, T_sk_ values in the dominant leg of a taller participant may also influence the results.

This study had some limitations. The study was applied to amateur male soccer players and included acute T_sk_ responses to the Wingate test. In the data extraction process, the quadriceps, hamstring, and gastrocnemius muscles were considered whole, and details about muscle parts (e.g., vastus lateralis, vastus medialis) were not presented. The groups for moderator variables were analyzed in a heterogeneous structure, and other moderator factors affecting T_sk_, such as gender and strength asymmetry, were not evaluated. Moreover, although the analyses were supported by Bayesian statistics, a limited number of participants were included in the study for subgroup analyses. This small sample size may have reduced the statistical power, particularly in detecting interaction effects and moderator-related differences. Finally, no evaluation was made on potential physiological parameters that may affect T_sk_. Additionally, the generalizability of the findings to elite-level soccer players remains limited, as the study focused solely on amateur athletes. The absence of performance-related outcome measures (e.g., sprint time, jump height, power output) and invasive physiological assessments (e.g., muscle biopsy, lactate concentration) also restricts the physiological interpretation of the thermal responses. Therefore, future studies can plan a similar study design based on the following suggestions: (i) with elite male soccer players, (ii) with elite or amateur female soccer players, (iii) with longer-term evaluations after the Wingate test (such as 1 hour, 4 hours and 8 hours after the Wingate test), (iv) with homogeneous comparison groups for moderator variables, (v) by evaluating different moderator variables, (vi) with the same study protocol including potential physiological parameters (lactate, inflammatory cytokines), (vii) incorporating objective performance metrics and muscle-specific assessments to better link Tsk changes with functional and structural adaptations, (viii) by utilizing dynamic time-series modeling techniques to capture transient thermoregulatory responses and state transitions [43], (ix) by integrating advanced machine learning algorithms such as Recurrent Neural Networks, Long Short-Term Memory networks, or Transformer models to model and classify thermographic time-series data.

## Conclusion

As a result, an increase in the dominant quadriceps T_sk_ may occur even after a 30-second maximum effort Wingate test in amateur male soccer players. This increase in T_sk_ may not be immediate after completing the Wingate test. The body fat percentage is a crucial moderating factor that influences the T_sk_ of amateur male soccer players. The T_sk_ of players with a lower body fat percentage may be assessed as higher than those with a higher body fat percentage. Therefore, field experts and coaches should consider body fat percentage when evaluating soccer players’ T_sk_ responses. If a soccer player with a high body fat percentage experiences inflammation or microtrauma, IRT may not detect this condition. Conversely, the T_sk_ of a soccer player with a low body fat percentage, who does not have inflammation or microtrauma, may still be assessed as elevated.

## Supporting information

S1 Appendix STROBE statement of the study. Checklist of reporting items recommended for observational studies.
(DOCX)

S2 Appendix Descriptive statistics of study data based on ROIs. Baseline and post-test values across different muscle groups and time points.
(DOCX)

S3 Appendix Descriptive statistics of study data based on moderator groups. Muscle activation outcomes stratified by dominant leg, smoking status, height, and body fat percentage.
(DOCX)

S1 Checklist Inclusivity in global research questionnaire. (DOCX)

## Abbreviations

STROBE: Strengthening the Reporting of Observational Studies in Epidemiology
TISEM: Thermographic Imaging in Sports and Exercise Medicine
OSF: Open Science Framework
T_sk_: Skin temperature
IRT: Infrared thermography
UV: Ultraviyole
Bayesian RM-ANOVA: Bayesian repeated measures ANOVA
BF_10_: Bayes factor for hypothesis testing
BF_incl_: Bayes factor for inclusion in the model comparison
ηp2: Partial eta squared
ANOVA: One-way repeated measures analysis of variance
