# Supplementary material for: Thermal imaging responses of lower-limb muscles following anaerobic testing in male soccer players: A time-series approach
Source: PLoS One. 2025 Oct 13;20(10):e0331102. doi: 10.1371/journal.pone.0331102 (PMC12517531; doi:10.1371/journal.pone.0331102)
Supplement: S2 Appendix — Baseline and post-test values across different muscle groups and time points. (DOCX) [file pone.0331102.s002.docx]

| **Appendix 2.** Descriptive statistics of study data based on ROIs. | | | | | |
| --- | --- | --- | --- | --- | --- |
| Variables | Baseline | Post-15 sec | Post-4 min | Post-8 min | Post-12 min |
| Left Quadriceps | 30.83±1.03 | 31.17 ± 1.20 | 31.16 ± 1.30 | 31.29 ± 1.37 | 31.38 ± 1.29 |
| Right Quadriceps | 30.76 ± 1.03 | 31.06 ± 1.15 | 31.14 ± 1.28 | 31.29 ± 1.25 | 31.38 ± 1.29 |
| Left Hamstring | 30.82 ± 1.08 | 31.03 ± 1.29 | 30.91 ± 1.19 | 30.95 ± 1.15 | 31.02 ± 1.20 |
| Right Hamstring | 30.78 ± 1.11 | 30.94±1.30 | 30.89 ± 1.18 | 30.92 ± 1.09 | 31.02±1.14 |
| Left Gastrocnemius | 30.36±0.95 | 30.53±1.01 | 30.51±0.99 | 30.65±0.96 | 30.73±0.92 |
| Right Gastrocnemius | 30.46 ± 1.03 | 30.55±1.06 | 30.51±0.97 | 30.73±0.96 | 30.61±1.23 |
| ***Legend.*** Post-15 sec; 15 seconds after the Wingate bicycle ergometer test; ROI: region of interest. | | | | | |
