# Supplementary material for: Thermal imaging responses of lower-limb muscles following anaerobic testing in male soccer players: A time-series approach
Source: PLoS One. 2025 Oct 13;20(10):e0331102. doi: 10.1371/journal.pone.0331102 (PMC12517531; doi:10.1371/journal.pone.0331102)
Supplement: S3 Appendix — Muscle activation outcomes stratified by dominant leg, smoking status, height, and body fat percentage. (DOCX) [file pone.0331102.s003.docx]

**Appendix 3.** Descriptive statistics of study data based on moderator groups.

|  | | | | | | | | | | | | | | | | |
| --- | --- | --- | --- | --- | --- | --- | --- | --- | --- | --- | --- | --- | --- | --- | --- | --- |
| Musle Groups | Time | Dominant leg status |  | Smoking status | |  | Body height status (cm) | | |  | Body fat status (%) | | | | |  |
|  |  | Dominant Leg Left dominant (n =7) Right dominant (n = 19) |  | Non-smoker (n = 20) | Smoker  (n = 6) |  | <=174 cm | 175 to 179 cm | >=180 cm |  | 5 to 10 | 11 to 14 | 15 to 20 | 21 to 24 | >24 |  |
| Left Quadriceps |  |  |  |  |  |  |  |  |  |  |  |  |  |  |  |  |
|  | Baseline | 30.39±1.36 |  | 30.94± 0.97 | 30.50±1.24 |  | 30.21±1.41 | 31.06±0.85 | 30.97±0.90 |  | 31.70±0.58 | 31.08±0.57 | 31.04±0.84 | 29.85±0.86 | 29.05±0.78 |  |
|  | Post-15 sec | 30.18±1.50 |  | 31.31±1.09 | 30.71±1.59 |  | 30.23±1.50 | 31.52±0.97 | 31.35±1.12 |  | 32.08±1.01 | 31.08±1.15 | 31.58±0.72 | 30.15±0.98 | 29.15±1.48 |  |
|  | Post-4 min | 30.49±1.67 |  | 31.26±1.19 | 30.81±1.68 |  | 30.47±1.51 | 31.37±1.12 | 31.37±1.37 |  | 32.12±1.27 | 31.52±1.29 | 31.30±0.77 | 30.40±1.09 | 28.70±0.71 |  |
|  | Post-8 min | 30.77±1.45 |  | 31.30±1.35 | 31.26±1.55 |  | 30.75±1.49 | 31.67±1.16 | 31.14±1.59 |  | 32.04±1.70 | 31.64±1.39 | 31.34±0.71 | 31.10±1.39 | 28.70±0.57 |  |
|  | Post-12 min | 31.00±1.53 |  | 31.38±1.19 | 31.38±1.71 |  | 31.25±1.54 | 31.56±1.30 | 31.21±1.23 |  | 32.24±1.24 | 32.00±1.25 | 31.26±0.86 | 30.90±1.52 | 29.25±0.35 |  |
| Right Quadriceps |  |  |  |  |  |  |  |  |  |  |  |  |  |  |  |  |
|  | Baseline | 30.88±1.03 |  | 30.77±1.08 | 30.73±0.95 |  | 30.47±1.26 | 30.97±0.84 | 30.67±1.20 |  | 31.70±0.72 | 31.14±0.66 | 30.83±0.87 | 29.77±0.67 | 29.10±0.57 |  |
|  | Post-15 sec | 31.34±0.97 |  | 31.10±1.16 | 30.95±1.21 |  | 30.52±1.35 | 31.27±0.96 | 31.17±1.29 |  | 32.06±1.13 | 30.94±1.01 | 31.41±0.76 | 29.97±0.85 | 29.35±0.07 |  |
|  | Post-4 min | 31.36±1.13 |  | 31.16±1.28 | 31.10±1.40 |  | 30.65±1.32 | 31.23±1.09 | 31.39±1.58 |  | 32.24±1.29 | 31.46±1.07 | 31.25±0.84 | 30.22±1.15 | 28.95±0.35 |  |
|  | Post-8 min | 31.46±1.19 |  | 31.25±1.28 | 31.41±1.26 |  | 30.90±1.40 | 31.53±1.09 | 31.22±1.46 |  | 32.04±1.70 | 31.52±1.17 | 31.27±0.78 | 31.05±1.45 | 29.45±0.64 |  |
|  | Post-12 min | 31.45±1.19 |  | 31.31±1.22 | 31.45±1.38 |  | 31.35±1.27 | 31.42±1.19 | 31.21±1.43 |  | 32.30±1.17 | 31.90±1.03 | 31.23±0.89 | 30.72±1.32 | 29.35±0.49 |  |
| Left Hamstring |  |  |  |  |  |  |  |  |  |  |  |  |  |  |  |  |
|  | Baseline | 30.45±1.35 |  | 30.85±1.04 | 30.75±1.31 |  | 30.73±1.45 | 31.11±1.40 | 30.47±0.84 |  | 31.64±0.89 | 31.06±1.08 | 30.84±1.05 | 30.30±0.65 | 29.20±0.57 |  |
|  | Post-15 sec | 30.31±1.62 |  | 31.15±1.21 | 30.66±1.61 |  | 30.62±1.51 | 31.42±1.03 | 30.77±1.48 |  | 32.20±0.82 | 30.96±1.37 | 31.06±0.91 | 30.87±1.29 | 28.55±0.12 |  |
|  | Post-4 min | 30.37±1.42 |  | 30.90±1.18 | 30.96±1.34 |  | 30.67±1.25 | 31.19±1.12 | 30.70±1.32 |  | 31.90±0.89 | 31.26±1.41 | 30.86±0.80 | 30.55±0.87 | 28.65±0.12 |  |
|  | Post-8 min | 30.72±1.26 |  | 30.91±1.18 | 31.08±1.14 |  | 31.05±0.92 | 31.17±1.15 | 30.55±1.34 |  | 31.76±0.95 | 31.32±1.31 | 30.82±0.86 | 30.82±0.94 | 28.95±1.20 |  |
|  | Post-12 min | 30.84±1.42 |  | 30.97±1.23 | 31.20±1.18 |  | 31.23±1.11 | 31.25±1.09 | 30.52±1.40 |  | 31.82±0.90 | 31.68±1.23 | 30.83±0.89 | 30.80±0.93 | 28.80±1.13 |  |
| Right Hamstring |  |  |  |  |  |  |  |  |  |  |  |  |  |  |  |  |
|  | Baseline | 30.92±1.06 |  | 30.79±1.14 | 30.75±1.09 |  | 30.83±1.42 | 31.00±1.06 | 30.42±0.98 |  | 31.56±0.90 | 31.00±0.97 | 30.82±1.25 | 30.20±0.48 | 29.30±0.28 |  |
|  | Post-15 sec | 31.21±1.13 |  | 31.05±1.26 | 30.56±1.48 |  | 30.68±1.40 | 31.37±1.05 | 30.49±1.51 |  | 32.04±1.02 | 30.78±1.44 | 30.95±1.00 | 30.85±1.29 | 28.75±0.07 |  |
|  | Post-4 min | 31.07±1.13 |  | 30.90±1.22 | 30.86±1.15 |  | 30.72±1.14 | 31.02±1.17 | 30.82±1.38 |  | 31.70±1.14 | 31.3±1.37 | 30.86±0.94 | 30.48±0.69 | 28.85±0.35 |  |
|  | Post-8 min | 31.00±1.09 |  | 30.88±1.13 | 31.05±1.03 |  | 31.23±0.90 | 30.99±1.03 | 30.59±1.34 |  | 31.68±0.91 | 30.96±0.93 | 30.90±0.98 | 30.90±0.99 | 29.10±1.41 |  |
|  | Post-12 min | 31.05±1.16 |  | 30.97±1.22 | 31.18±0.90 |  | 31.37±1.06 | 31.11±1.03 | 30.64±1.40 |  | 31.68±0.93 | 31.52±1.11 | 30.93±0.96 | 30.75±0.95 | 29.15±1.62 |  |
| Left Gastrocnemius |  |  |  |  |  |  |  |  |  |  |  |  |  |  |  |  |
|  | Baseline | 30.34±1.00 |  | 30.43±.99 | 30.17±0.87 |  | 30.42±1.20 | 30.65±0.93 | 29.91±0.68 |  | 30.66±1.08 | 30.56±1.05 | 30.51±1.00 | 29.67±0.34 | 29.85±0.92 |  |
|  | Post-15 sec | 30.16±1.25 |  | 30.61±1.00 | 30.30±1.11 |  | 30.32±1.19 | 30.87±1.03 | 30.20±0.81 |  | 31.18±0.98 | 30.36±1.03 | 30.54±0.87 | 30.67±1.22 | 29.10±0.42 |  |
|  | Post-4 min | 30.33±1.23 |  | 30.52±0.98 | 30.48±1.12 |  | 30.22±1.26 | 30.76±0.98 | 30.38±0.83 |  | 30.62±1.19 | 30.84±0.99 | 30.78±0.87 | 30.10±0.70 | 28.95±0.07 |  |
|  | Post-8 min | 30.69±1.12 |  | 30.61±0.98 | 30.78±0.98 |  | 30.82±0.89 | 30.87±1.08 | 30.20±0.77 |  | 30.90±0.91 | 30.80±1.10 | 30.76±0.93 | 30.35±1.09 | 29.75±1.06 |  |
|  | Post-12 min | 30.67±1.02 |  | 30.71±0.95 | 30.78±0.93 |  | 30.95±0.73 | 30.94±0.96 | 30.25±0.92 |  | 30.94±1.00 | 31.18±0.93 | 30.70±0.93 | 30.52±0.72 | 29.65±092 |  |
| Right Gastrocnemius |  |  |  |  |  |  |  |  |  |  |  |  |  |  |  |  |
|  | Baseline | 30.51±1.02 |  | 30.56±1.13 | 30.15±0.63 |  | 30.55±1.28 | 30.61±0.97 | 30.20±1.03 |  | 30.56±1.56 | 30.92±0.97 | 30.64±0.93 | 29.65±0.34 | 29.90±0.70 |  |
|  | Post-15 sec | 30.30±1.26 |  | 30.68±1.06 | 30.13±1.06 |  | 30.35±1.34 | 30.79±1.04 | 30.36±0.93 |  | 31.08±1.23 | 30.62±1.18 | 30.56±0.81 | 30.55±1.27 | 29.10±0.42 |  |
|  | Post-4 min | 30.31±1.03 |  | 30.57±1.00 | 30.32±0.94 |  | 30.27±1.11 | 30.64±0.97 | 30.50±0.97 |  | 30.54±1.28 | 30.80±1.05 | 30.75±0.90 | 30.15±0.52 | 29,15±0.07 |  |
|  | Post-8 min | 30.88±1.08 |  | 30.76±0.99 | 30.65±0.94 |  | 31.22±080 | 30.82±0.97 | 30.25±0.95 |  | 30.92±0.81 | 30.74±1.25 | 30.75±087 | 30.97±1.00 | 29.75±1.48 |  |
|  | Post-12 min | 30.79±0.94 |  | 30.57±1.34 | 30.75±0.87 |  | 31.03±0.91 | 30.58±1.58 | 30.34±0.85 |  | 30.92±0.80 | 31.16±1.09 | 30.49±1.54 | 30.27±0.94 | 29.75±1.63 |  |
